# Antimicrobial stewardship in community pharmacy: a full review of online educational resources

**DOI:** 10.1093/jacamr/dlag101

**Published:** 2026-06-12

**Authors:** Sejal Parekh, Naomi Fleming

**Affiliations:** Primary care, Community, Vaccinations and Screening Directorate NHS England, London SE1 8UG, UK; AMR Programme, Medical Directorate, NHS England, London SE1 8UG, UK

## Abstract

**Background:**

Significant progress has been made in embedding strong antimicrobial stewardship (AMS) practices within community pharmacy teams as their roles expand to meet growing primary care appointment demand. To maintain this momentum, it is essential that registered community pharmacy professionals, whether newly entering the workforce or refreshing their skills, have access to high quality online learning resources that support the development, renewal and expansion of their AMS knowledge.

**Aim:**

To identify and assess the available online antimicrobial stewardship training resources suitable for community pharmacy teams in England.

**Methods:**

A search was conducted to identify easily accessible online antimicrobial educational resources specifically designed for, or suitable for, community pharmacy professionals

**Results:**

Twenty online resources were identified, of which 12 were selected for evaluation. The resources ranged from being suitable for novices to advanced learners with specific learning needs.

**Conclusions:**

Although there are few education resources created specifically for community pharmacy professionals, a wide range of online materials are suitable and can provide a strong foundation in antimicrobial knowledge and support further learning at the learner’s own pace. Targeted resources can help address individual learners’ questions, while many of the available materials offer practical applications relevant to both community pharmacy and broader primary care settings. Since many of these resources are open to all healthcare professionals, they standardize knowledge across the workforce and support better integration of community pharmacy within the wider NHS system.

## Introduction

Antimicrobial resistance (AMR) continues to pose a significant global health challenge, affecting individuals both directly—through infections that become increasingly difficult to treat—and indirectly by placing substantial pressures on healthcare systems.^1^ The impact on human health is well documented; in fact it is estimated bacterial AMR is responsible for 1.27 million deaths globally and contributed to 4.95 million deaths in 2019. In England, rising demand for primary care services has intensified pressure on general practice, resulting in more patients seeking support from community pharmacies for the management of acute, self-limiting infections.^2,3^ Concurrently, recent policy developments have broadened the clinical and advisory responsibilities of community pharmacists, positioning them as first contact healthcare professionals for a growing range of conditions. As a result, members of the public are increasingly relying on community pharmacists for timely assessment, evidence-based advice, and reassurance when managing common infections and minor ailments.^4–7^

Themes 1 and 2 of *Confronting Antimicrobial Resistance: 2024 to 2029* emphasize the need to reduce unnecessary exposure to antimicrobials and to optimize their use, respectively.^8^ Achieving these ambitions requires meaningful engagement with the public, and community pharmacy teams are central to this effort. To fulfil this role effectively, pharmacy professionals must have access to high quality training and evidence-based resources, and ongoing educational support. This is essential not only for developing their own competencies in antimicrobial stewardship (AMS) but also for enabling them to communicate accurate, accessible and practical information to the populations they serve.

Since 2020, the sector has been trained in antimicrobial stewardship via the Pharmacy Quality Scheme, receiving both infection control and antimicrobial stewardship training.^4–7^ Several resources have already been used nationally across the sector, helping to build a strong and sustainable foundation in AMS and ensuring that community pharmacy teams are equipped to support stewardship efforts and protect the future effectiveness of antimicrobials as well as practically using the TARGET toolkit resources to promote structured conversations with patients.^4–7^

The indicative curricula developed for undergraduate pharmacy was published in 2025; however, its implementation into pharmacy courses is not mandatory.^9^ It is therefore not guaranteed that pharmacy graduates (graduating as prescribers in 2026) will have the skills and knowledge to manage patients requiring understanding of antimicrobial stewardship and/or their infections.

It is therefore important for registered community pharmacy professionals either entering the profession or requiring a refresh of their skills to be able to access suitable online education resources to learn, renew and update their AMS knowledge. This paper assesses online training materials on AMR and AMS suitable for community pharmacy professionals in England.

### Aim:

The aim of this review is to identify and assess the available online antimicrobial stewardship training resources suitable and easily accessible for community pharmacy professionals in England.

## Methods

This review used the Arksey and O’Malley framework, which consisted of six stages designed to answer the research question.^10^

### Stage 1. Identifying the research question

This assessment sought to review online educational resources in AMR and AMS suitable for community pharmacy professionals and answer the following question:

*What easily accessible online educational resources are suitable for pharmacy professionals practising in community pharmacy that provide training in antimicrobial resistance and promote antimicrobial stewardship?*

The following objectives were proposed:

- Identify educational online resources that could be used for training community pharmacy professionals in antimicrobial stewardship.
- Select and assess the identified online educational resources against the research questions.
- Analyse and compare characteristics of educational resources.
- Evaluate each training resource.

### Stage 2. Identifying relevant resources

Resources from well-known pharmacy AMS and AMR educators were screened for suitability for community pharmacy professionals. These included the Centre for Pharmacy Postgraduate Education (CPPE), treat antibiotics responsibly, guidelines education and tools (TARGET), BSAC, Primary Care Pharmacy Association, PrescQIPP, Royal Pharmaceutical Society (RPS), UK Clinical Pharmacy Association (UKCPA), e Learning for Health (eLFH), Antibiotic Guardian, Scottish Antimicrobial Pharmacy Group and Public Health Wales and an online search of grey literature.

An online ‘grey literature’ search was conducted using the terms ‘Community pharmacy antimicrobial stewardship training’ and ‘Community pharmacy antimicrobial resistance training’.

### Stage 3. Selection of resources for review

A two-person methodology was applied for resource selection. N.F. searched and listed all potential resources independently. These were then checked and organized by S.P. to ensure no resources were missing and decide whether they were suitable for community pharmacy professionals. Each author assessed whether the training resource met the initial research questions, checking the accessibility of the resource and potential costs to access/complete the training. The authors then collectively synthesized their individual findings, reviewing and assessing each training resource to reach a consensus to determine whether the resource should be included in the review. If the resource was not included, an explanation/justification is provided (Table 1). Suitability was assessed by the authors completing the training and determining whether it was predominantly intended for a community pharmacy audience and/or whether it could be appropriately applied within a community pharmacy or primary care setting. This assessment was based on the content of the learning, for example, ensuring it did not reference specialist services or indicate that it was designed for secondary or tertiary care environments.

**Table 1.** Training and education resources excluded

Antimicrobial stewardship and resistance training resource	Link to training	Rationale for exclusion
Providing NHS and public health pharmacy services NHS Pharmacy First Service By CPPE	https://www.cppe.ac.uk/services/pharmacy-first/#navTop	This was excluded as it focuses on provision of the pharmacy first service that requires community pharmacies to hold consultations that give advice and NHS-funded treatment (via Patient Group Directions), where appropriate for seven common conditions (following clinical pathways), it is not an educational resource on AMR or AMS.
Antimicrobial Resistance (AMR) and ‘One Health': Current Challenges, Regulatory Frontiers and Future Solutions Training Course	https://ipi.academy/product/details/2800/antimicrobial-resistance-amr-and-one-health-current-challenges-regulatory-frontiers-and-future-solutions?utm_source=google&utm_medium=cpc&utm_campaign=wrd-ga-Pmax-top-courses&utm_id=22264184522&utm_term=&gad_source=1&gad_campaignid=22257641160&gclid=EAIaIQobChMIiaj_rYPXkgMV4plQBh2_DCpuEAAYASAAEgLZU_D_BwE	This course allows participants to explore the regulatory landscapes governing AMR, discover innovative solutions and understand the challenges AMR poses across different sectors. 1 day live online training. Excluded as unable to access as cost £649.
UK Clinical Pharmacy Association (UKCPA) infection community	https://portal.ukclinicalpharmacy.org/login	This portal contains resources, presentations and webinars on AMS, AMR and related specialist topics. There is also a forum for discussion and advice from peers. This resource was excluded as the majority of content is aimed at secondary care and a paid subscription is needed to access the portal and content.
Learning @ Wales	https://learning.nhs.wales/mod/page/view.php?id=15348	This learning platform was excluded as registration requires you have an NHS Wales email account for access to the platform.
Antimicrobial Stewardship & resistance for community pharmacy e-learning hosted on Health Education and Improvement Wales (HEIW)	https://ytydysgu.heiw.wales/courses/c20cc32d-53b9-4658-8df4-9d16802313ca	This particular resource was excluded as registration requires you have an NHS Wales email account to access the e-learning.

### Stage 4. Data extraction

A standardized data extraction form (Figure 2) was used to assess the characteristics of the selected resources, where the following information was evaluated:

- Anticipated duration to complete.
- Accreditation/provision of a certificate.
- Language.
- Reusable/downloadable.
- Access.
- Resource type.
- Source of resource.
- Focus of topic/resource.
- Target audience.
- Relevance/applicability.
- Setting of resource.

### Stage 5. Data summary and synthesis of results

Relevant information was collated for each resource including learning type (narrated video, quizzes, case studies) and key messages that the resources expressed. The authors also outlined where different resources may complement and/or overlap and cover similar ground. The authors have indicated the level of knowledge gained from the different courses to help learners identify the most suitable option or combination of options for their needs.

## Results

**Figure 1.**
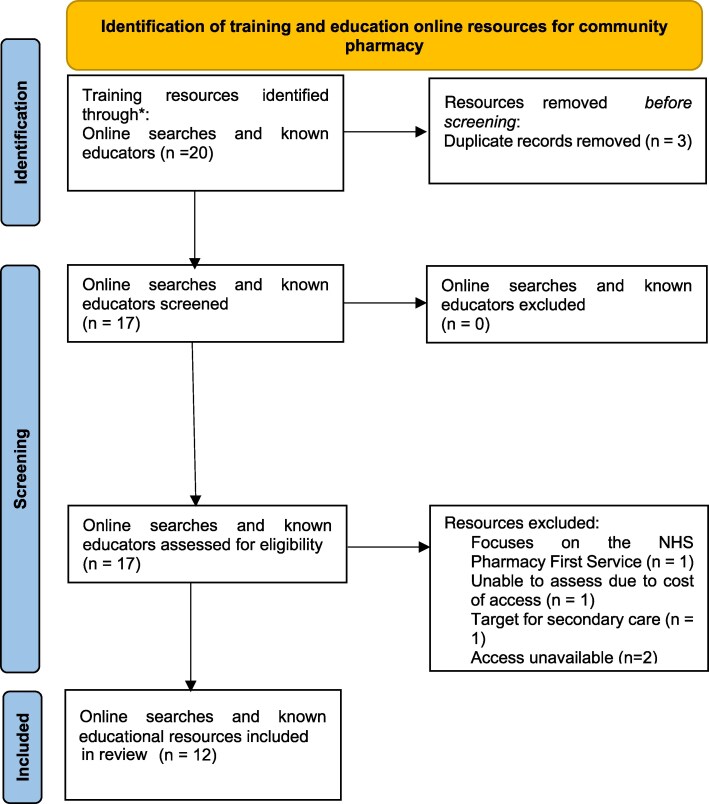
PRISMA flow diagram of identification and selection of online training and education resources in antimicrobial stewardship for community pharmacy.

A total of 20 educational resources were identified. Eight of these resources were excluded- See *Figure 1* PRISMA flow chart. Full details for resources that were excluded can be found in Table 1.

**Figure 2.**
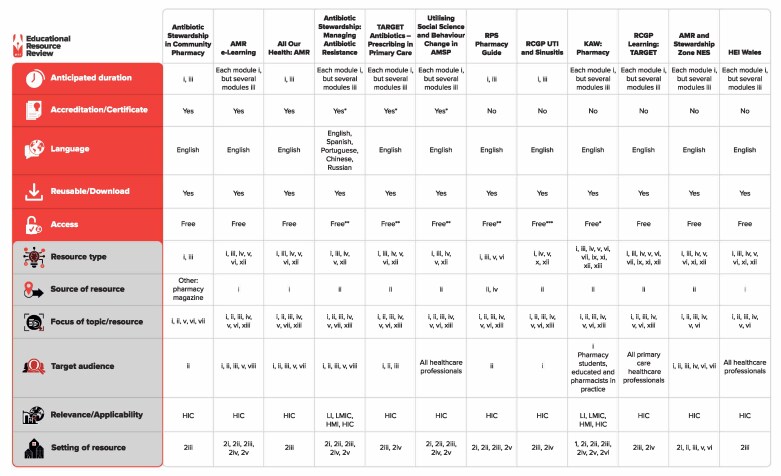
General characteristics of resources evaluated in this review. Antibiotic Stewardship in Community Pharmacy: https://www.pharmacymagazine.co.uk/cpd-modules/antibiotic-stewardship-in-community-pharmacy;^11^ AMR e-Learning, Antimicrobial Resistance (AMR) (e-Learning for Healthcare): https://portal.e-lfh.org.uk/Component/Details/411808;^12^ All Our Health: AMR, All Our Health: Antimicrobial Resistance: https://portal.e-lfh.org.uk/Component/Details/571263;^13^ Antimicrobial Stewardship: Managing Antibiotic Resistance: https://www.futurelearn.com/courses/antimicrobial-stewardship;^14^ TARGET Antibiotics—Prescribing in Primary Care: https://www.futurelearn.com/courses/target-antibiotics;^15^ Utilizing Social Science and Behaviour Change in AMSP, Utilizing Social Science and Behaviour Change in Antimicrobial Stewardship Programmes: Improving Healthcare: https://www.futurelearn.com/courses/target-antibiotics;^16^ RPS Pharmacy Guide, RPS Pharmacy Guide: Antimicrobial Resistance and Stewardship Resources: https://www.rpharms.com/resources/pharmacy-guides/antimicrobial-resistance-and-stewardship-resources;^17^ RCGP UTI and Sinusitis, Royal College of General Practitioners (RCGP): Common infections in primary care—management of urinary tract infections and sinusitis: https://elearning.rcgp.org.uk/course/view.php?id=333;^18^ KAW: Pharmacy, Keep Antimicrobials Working: Pharmacy: https://bsac-kaw.co.uk/pharmacy/;^19^ RCGP Learning: TARGET, RCGP Learning: TARGET Antibiotics Toolkit Hub: https://elearning.rcgp.org.uk/course/view.php?id=553;^20^ AMR and Stewardship Zone NES, Antimicrobial Resistance and Stewardship Zone National Education Scotland: https://learn.nes.nhs.scot/75514;^21^ HEI Wales, Health Education and Improvement Wales: https://heiw.nhs.wales/education-training/support/campaigns/antimicrobial-resistance-and-stewardship/.^22^ Anticipated duration: (i) short 4 h; (ii) long >4 h; (iii) self-paced. Accreditation/Certificate: Yes/*Yes with paid subscription/No/Not applicable; Language; Reusable/Download: yes/no/not applicable; access: free; **Free to review and learn but no certificate will be provided without subscription; ***Free to RCGP Members; cost; not applicable. Resource type: (i) website online reading material and other resource; (ii) website primarily aimed at news items; (iii) online/distance learning courses [massive open online course (MOOC), unfacilitated courses, online modules]/community of practice; (iv) webinars, video, online lectures, podcasts, animation video, maps, photos; (v) clinical practice antimicrobial stewardship materials: PDFs, PowerPoints, newsletters, infographics, pamphlets, e-portfolios, workbooks; (vi) guidelines, policies, handbooks; (vii) material from face-to-face non-e-learning courses (programmes, teaching materials etc. from workshops, lectures, seminars); (viii) e-books via apps; (ix) public, media, political awareness and engagement materials; (x) commercial advertising (TV, radio, film, social media); (xi) evidence: by systematic reviews/meta-analysis in relation to antimicrobial resistance; (xii) datasets, compelling/illustrative case studies on antimicrobial stewardship; (xiii) patient stories. Source of resource: (i) governments; (ii) professional societies; (iii) universities/higher education institutes; (iv) healthcare facilities; (v) WHO; (vi) industry; (vii) insurance companies; (viii) non-governmental organizations (NGOs). Focus of resource: (i) principles/practice of prudent prescribing; (ii) antimicrobial stewardship principles/practices; (iii) guidelines/policies/pathways for syndrome management of infections; (iv) infection prevention/control; (v) implementation/behaviour change; (vi) evaluation/measurement; (vii) evidence gathering. Target audience: (i) doctors; (ii) pharmacists; (iii) nurses/midwives; (iv) non-medical managers; (v) public health; (vii) laboratory; (viii) infection prevention practitioners. Relevance/applicability: LIC, low-income country; LMIC, low- and middle-income country; HMIC, high- and middle-income countries; HIC, high-income country. Setting of resource: (1) pre-service (university, higher education institution); (2) service: (i) hospital; (ii) outpatient clinic; (iii) community/general practice; (iv) long-term care facility/nursing home; (v) hospital and ambulatory; (vii) other.

The PRISMA flow chart (Figure 1) shows the 12 online resources evaluated in this study ranging from stand-alone modules, on-line courses with two or more modules and AMR/AMS education repositories containing suitable resources for community pharmacy professionals. They are free for healthcare professionals and acesss does not require an individual paid membership fee. Each of the 12 educational online resources could be used for training community pharmacy professionals in antimicrobial stewardship and therefore meet the study objectives. Figure 2 summarises the resources evaluated.

### Antibiotic stewardship in community Pharmacy

This training module focuses on antibiotic stewardship within community pharmacy and is suitable for pharmacy teams undertaking continuing professional development.^11^ It offers a concise overview and foundational understanding of AMR and AMS. It is particularly suitable for those with no prior AMS experience, and its bite-sized format allows it to be completed conveniently during breaks or lunch periods.

Its strengths include being specifically tailored to a community pharmacy audience and taking only around 20 min to complete. Launched in May 2025, it contains up to date information. However, it remains an introductory resource, is available only in English, and is not accredited by an official body.

### Antimicrobial resistance (e-learning for healthcare)

This training package consists of the following modules^12^:

- Antimicrobial Resistance Toolkit.
- Introduction to antimicrobial resistance.
- Antibiotic Review Kit.
- Antimicrobial Stewardship for Community Pharmacy.
- Case studies.
- Films.
- Antimicrobial prescribing for common infections.

The majority of the training is intended for all healthcare staff; however, there is one module specifically designed for community pharmacy. The ARK module is focused on secondary care, while the remaining modules are relevant to all community pharmacy professionals.

The strengths of this training include its modular structure, allowing learners to complete the modules in any order or as standalone components. It is freely accessible and does not require paid professional membership (an NHS email address is required, which all healthcare professionals in England can access). Knowledge quizzes and checkpoints help assess understanding, and formal assessments can be used as evidence of continuing professional development (CPD). The inclusion of multimedia resources supports different learning styles. Most of the training is tailored to the UK healthcare system, which may limit its relevance in countries with different structures or resource levels. The content was last updated in January 2022, meaning the community pharmacy module does *not* include developments introduced since then such as the NHS Pharmacy First Service or more recent progress in community pharmacy and its role in antimicrobial use and stewardship. The training is available only in English. It is well suited to individuals aiming to enhance their AMS competency and provides a strong foundation for those with no prior knowledge, supporting them to achieve a moderate level of understanding and proficiency.

### All our health: antimicrobial resistance

This bite-sized session provides health and care professionals with an overview of AMR, including key evidence, data, and signposts to trusted resources that support illness prevention, health protection and wellbeing promotion.^13^

Designed for a broad healthcare audience, it offers consistent messaging across professions, is quick to complete, and is relatively recent (October 2024). The training is accessible to all and does not require paid professional membership; however, an NHS email address is needed, which all healthcare professionals in England can obtain.

This resource is not an in-depth educational programme on AMR or AMS. Its content is primarily tailored to the UK healthcare system, which may limit its relevance in countries with significantly different structures or resources. Additionally, it does not include a dedicated section for community pharmacy professionals and is available only in English.

### Antimicrobial stewardship: managing antibiotic resistance

Designed for healthcare professionals, this 6-week course (approximately 3 h per week) provides learners with a comprehensive understanding of safe, high quality antibiotic use.^14^ It is delivered periodically to international cohorts, where it explores what antibiotic resistance is and why the World Economic Forum lists it alongside terrorism and climate change on its global risk register.

The course covers:

- Antibiotic resistance and its global impact.
- The relationship between resistance and prescribing practices.
- Principles of antimicrobial stewardship and its implementation in hospital settings.
- The importance of measurement in stewardship and how it improves antibiotic prescribing.
- The role of novel diagnostics in clinical decision-making.
- The value of Behaviour Change Science in improving prescribing.
- Examples of successful stewardship initiatives from around the world.

This course is accredited by the Royal College of Pathologists and includes interactive learning through message boards with educators and global, cross sector colleagues. Its multimedia resources support diverse learning styles, and the training is available in multiple languages. It is particularly suited to learners with a specialist interest in AMR and AMS. It is focused on a secondary setting but contains theory to understand the foundation of AMS as well as behavioural change science that could be applied to different settings. This would be ideal for community pharmacy staff wanting further knowledge or having a specific in depth interest in AMS, but use within community pharmacy is otherwise limited.

The course does have some limitations: it is only available free of charge during specific cohort periods, and learners must pay if they wish to receive certification. It also requires a significant time commitment over a relatively short six-week span. While the content is thorough, it is not specifically tailored to primary care or community pharmacy settings, which may limit its relevance for community pharmacy professionals. Additionally, the course has not been updated recently.

### TARGET antibiotics—prescribing in primary care

This 5-week course (requiring a commitment of approximately 1 h per week) explores several aspects of antibiotic prescribing in primary care, from prescribing for urinary tract infections to managing patient expectations.^15^

The course covers:

- Introduction to antimicrobial resistance in primary care.
- Prescribing for urinary tract infections.
- Assessing the need for antibiotics.
- Managing patient expectations and back-up/delayed prescribing.
- Common practice approaches.

This training package is designed for a broad healthcare audience, ensuring consistent messaging across different professional groups. The course is accredited by the Royal College of Pathologists; it offers interactive learning through message boards with educators and peers the multimedia resources support diverse learning styles.

The course is ideal for those working in primary care or community pharmacy that have an interest in AMR and AMS. However, it is important to note that it runs for free only during specific cohort periods throughout the year, and certification is available to paying participants only. While the content is in depth and highly relevant to primary care, it is not specifically tailored to community pharmacy. However, it could provide a sound background in understanding the UTI clinical pathway for the NHS Pharmacy First Service. The course is delivered in English only.

### Utilizing social science and behaviour change in antimicrobial stewardship programmes improving Healthcare

This 2-week course (requiring a commitment of approximately 3 h per week) builds on Week 5 of *Antimicrobial Stewardship: Managing Antibiotic Resistance*,^14^ although it can also be taken as a standalone learning opportunity.^16^

The course covers the following:

- How social science can support improvement in healthcare.
- Understanding who needs to do what, differently, for which patients to achieve better outcomes.
- Identifying barriers and facilitators to change.
- Using social science frameworks to design effective improvement interventions.

Accredited by the CPD Certification Service, this course provides opportunities to interact with educators and fellow learners via message boards, enabling global exchange of experiences and insights from different healthcare systems. Multimedia resources cater to a variety of learning styles.

The course focuses on behaviour change and how behavioural science can be harnessed to improve antimicrobial use. It is suited to anyone interested in healthcare improvement or behaviour change science across any healthcare sector, particularly those looking to apply these principles to AMR and AMS.

The course is available for free during designated cohort periods throughout the year, with certification offered only to paying participants. Although not specifically tailored to community pharmacy, it is suited and relevant for community pharmacy professionals.

### Royal pharmaceutical society: pharmacy guide—antimicrobial resistance and stewardship

There is a freely accessible quick guide offering 10 top tips on how to tackle AMR and promote safer antimicrobial use.^17^ It includes links to trusted external resources and serves as a practical introduction to implementing AMS as a community pharmacy professional.

Additional, more detailed sections are available to Royal Pharmaceutical Society (RPS) members, covering the following:

- Prescribing antimicrobials.
- Dispensing antimicrobials—delayed prescriptions and clinical checks.
- Educating people on the safe and effective use of antimicrobials.
- Advising on self-limiting conditions and self-care.
- Counselling when dispensing or supplying antimicrobials.
- Providing written resources and signposting to further advice.
- Training and education for pharmacy teams.
- Further information.

These resources are community pharmacy-focused, recently updated (September 2024) and include clear, actionable steps to support AMS implementation in practice. However, access to the full resource is restricted to RPS members, and all content is available in English only.

### Royal college of general practitioners (RCGP): common infections in primary care—management of urinary tract infections and sinusitis

This set of three screencasts outlines the latest evidence-based approach to the assessment and management of urinary tract infections (UTIs) and sinusitis.^18^ The two UTI screencasts distinguish between the different approaches that should be adopted depending on the age of the patient and particularly offers guidance on the role of dipstick and urine culture. The sinusitis screencast considers all ages and offers guidance on distinguishing between bacterial and viral sinusitis and when antibiotics should be issued.

A key strength of this training is the format of short, 5-min screencasts that are easy to fit into a busy working day. The content is relevant to Community Pharmacy First services, is CPD accredited, and was most recently reviewed in December 2025. Although designed with a general primary care focus, the material remains applicable to community pharmacy professionals.

The training does not provide direct education on AMR or AMS and is not specifically tailored to community pharmacy settings, although the clinical content is still useful for those working in these roles. While the resources are free to access, registration with the RCGP is required. Finally, the training is available only in English and is aligned with UK primary care practice.

### Keep antimicrobials working: pharmacy

This resource is a repository of AMS education and training resources, national policies, guidelines and relevant publications for educators and undergraduate pharmacy students as well as those wishing to learn more about AMS, AMR or specific infections.^19^ The repository can be searched by AMR/AMS curricula domain including Antibiotic prescribing and stewardship, Antimicrobials and antimicrobial resistance, infection prevention and control, interprofessional collaborative practice, person centred care and vaccine uptake.

The content can also be filtered by resource type depending on preferred learning style, for example, animations, apps, games, e-books, e-learning, revision guides, videos and webinars. This makes this resource very comprehensive and flexible in application depending on the learners needs. There are also KAW resources for medics, dentists and nurses who share the appropriate materials ensuring consistency of education and training across the professional undergraduate training programmes.

Many of the topics offer concise, focused information, with ‘speed learning’ segments that can be completed in around one minute. While the content is not tailored specifically to community pharmacy practice, much of the learning can be applied. The learning is in English and focuses on AMS in the UK. Overall, it is a useful, flexible tool for quickly gaining insight into specific clinical topics. It is however a large repository of information and could be overwhelming for the individual learner but could be used to search for learning materials and address identified specific knowledge gaps.

### RCGP learning: TARGET antibiotic toolkit hub

This hub provides a comprehensive collection of resources designed to support primary care clinicians in championing and implementing antimicrobial stewardship (AMS) activities.^20^ The materials can also contribute to CPD and revalidation requirements, making it a practical tool for ongoing professional development. A dedicated section is available for community pharmacy teams, with focused content on respiratory tract infections (RTIs) and urinary tract infections (UTIs). Additional training materials are aimed at the wider primary care workforce but remain valuable for gaining broader clinical insight. The hub also offers free recorded webinars, accessible on demand through the website. The learning is free to access, delivered in English, and aligned with UK health priorities, making it a useful and relevant resource for community pharmacy professionals seeking to strengthen their AMS knowledge and practice.

### NHS education for Scotland, TURAS learn: antimicrobial resistance and stewardship Zone

This Scottish resource is for health and social care staff. It includes a knowledge and skills framework covering 6 domains and learning resources for each of these domains.^21^ Domain 1: Foundations—awareness of AMR and AMS,^23^ Domain 2: Prevention and control of infection,^24^ Domain 3: Diagnosis of infection and clinical decision-making,^25^ Domain 4: Diagnostic stewardship,^26^ Domain 5: Treatment and management—appropriate use of antimicrobials,^27^ Domain 6: Communication, education, collaborative practice and management.^28^ Some of the resources are primary care focused and there are several designed for community pharmacy. There are different types of resources available for different learning styles and various lengths of training from a few minutes to several hours. It is a structured learning environment with key resources under each of the domains ensuring a standard level of knowledge in all the domains.

Learning resources have been created following clinical guidelines for Scotland. These might be different to guidelines in other UK nations or countries. The resources are only available in English and a log in to Turas is required ( this is free).

### Health education and improvement Wales

This contains a range of resources including international campaigns such as World Antimicrobial Awareness Week and European Antibiotic Awareness Day.^22^ There are relevant sections for the provisions in Wales where the understanding and purpose of a back-up antibiotic prescribing could be applied within England. The strength of this training is the inclusion of short videos, which are ideal during workplace activities. The learning is free to access, delivered in English and Welsh covering different AMS topics than the other resources, making it a valuable addition, specifically adding insight into World and European campaigns as well as understanding the purpose of delaying prescriptions.

## Discussion

There is a wide range of AMR and AMS educational resources that can be adapted for community pharmacy professionals. These materials span from introductory content—such as Antibiotic Stewardship in Community Pharmacy—to more structured learning that build a strong foundation in AMR and AMS, including courses like AMR on eLearning for Healthcare. Once this foundational knowledge is in place, additional training resources can be used to deepen understanding and support learning for more specific clinical scenarios.

Most available resources align with UK national priorities and strategies, reflecting the UK’s strong focus on maintaining high standards in antimicrobial stewardship. With appropriate adaptation, many of these materials also have the potential to be used in international contexts.

As community pharmacies are often the first point of contact for patients, they play a significant public health role in AMR prevention. AMS education supports pharmacy teams to deliver effective self-care advice, provide clear and consistent messages on antibiotic use aligning with other primary care healthcare professionals and reducing unnecessary demand for antibiotics. This makes investment in pharmacy-focused AMS training particularly important.

Many AMR and AMS educational resources are designed for a broad range of primary care practitioners and are valuable for community pharmacy teams. This multidisciplinary approach to learning enhances clinical knowledge, encourages interprofessional collaborative practice and supports integration of community pharmacy professionals into the wider primary care workforce.

Further training that incorporates behavioural science, communication strategies and supports shared decision-making—particularly around managing patient expectations for antibiotics—can enhance practice by helping clinicians strike the right balance between reassurance, effective safety netting and appropriate antibiotic use.

It is well known that potential barriers such as time pressures in busy community pharmacies can limit learning. However, a lot of the resources are flexible, modular and bitesize, which helps to address these challenges.

Ultimately, the choice of training will depend on individual learners and their preferred pace, but the breadth of resources available offers ample options for community pharmacy professionals. These materials are both relevant and practical, aligning well with managing common scenarios—patients presenting with RTIs and UTIs as well as current NHS services such as Pharmacy First—and can be readily applied in day-to-day practice.

### Conclusions

The current training provisions can be used to educate and reinforce AMS knowledge for new and existing community pharmacy teams. There is a variety of training resources, many of which were not initially designed for community pharmacy but are suitable and easily accessible by community pharmacy teams. A lot are flexible in being modular and/or bitesize.

As the role of community pharmacy continues to expand with services such as Pharmacy first and independent prescribing, there will be differing needs for education on AMS/AMR based on the role of the pharmacy professional and the services they deliver. The knowledge and skills required are therefore based on a personal analysis of need.

The range of resources currently available is appropriate; however, a single, centralized platform that signposts community pharmacy teams to these resources would be beneficial.

It is important for resources to keep pace with changing roles of community pharmacy professionals and have options suitable for novice to experienced practitioners encompassing the variety of roles conducted by community pharmacy in England. Embedding AMS education into ongoing professional development supports safe and appropriate prescribing, improves management of common infections such as RTIs and UTIs, as well as aligning with national ambitions.
